# Molecular dynamics simulations on ε-CL-20-based PBXs with added GAP and its derivative polymers†

**DOI:** 10.1039/c7ra13517c

**Published:** 2018-01-29

**Authors:** Yingying Lu, Yuanjie Shu, Ning Liu, Xianming Lu, Minghui Xu

**Affiliations:** Xi'an Modern Chemistry Research Institute Xi'an Shaanxi 710065 China Syj1204172675@163.com

## Abstract

Molecular dynamics simulations have been employed to study the ε-CL-20-based PBXs under COMPASS force field. ε-CL-20 was chosen as the base explosive due to its higher energy, density and detonation performance than conventional explosives. Four polymers, GAP, GAP-NH_2_, GAP-NO_2_ and GAP-NH_2_-NO_2_ were added into the ε-CL-20(001) crystalline surface to build the PBX models. The cohesive energy densities (CEDs), elastic coefficients, isotropic mechanical properties (Young's moduli, bulk moduli, shear moduli, Poisson's ratio, Cauchy pressure and *K*/*G*) and initiation bond length distribution were studied. It turned out that the CEDs order was A1 < A4 < A3 < A2 < A. The mechanical properties of pure ε-CL-20(001) were effectively improved by building PBX models. System A3 showed better comprehensive mechanical properties than the other three PBXs. A study on the initiation bond length distribution showed that the *L*_max_ and *L*_ave_ of N–NO_2_ increased with increasing temperature and they were related to the sensitivity of the explosives. The order of *L*_max_ was A3 < A4 < A2 < A1 < A, which indicated that the PBXs owned lower sensitivity than system A. These studies are thought to provide guidance for further research on the application of GAP and its derivative polymers. Meanwhile, they are meaningful for the studies on ε-CL-20-based PBXs.

## Abstract 2

Molecular dynamics simulations have been employed to study the ε-CL-20-based PBXs under COMPASS force field.

## Graphical abstract

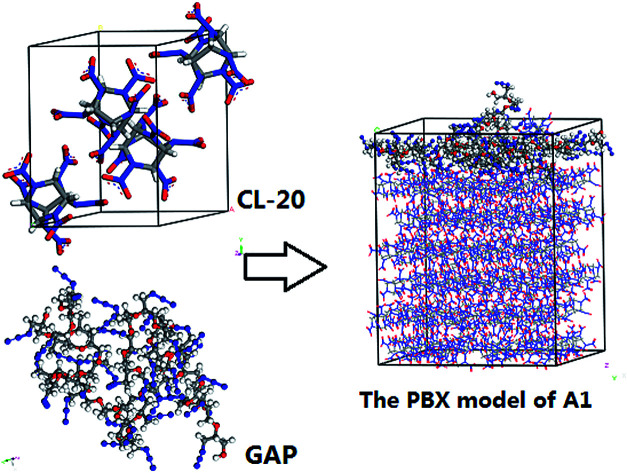

## 1. Introduction

**Fig. 1:**
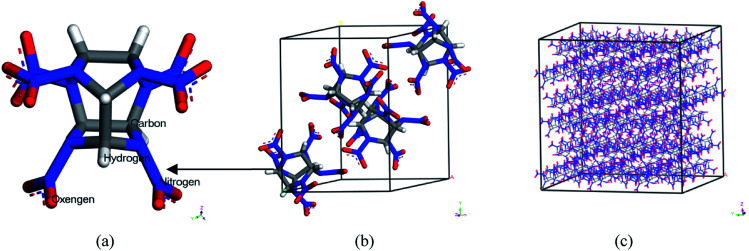
The molecular structure of CL-20 (a), single crystal (b) 4 × 3 × 3 supercell (c).

Polymer-bonded explosive (PBX) is a kind of composite explosive with perfect detonation performance, such as high energy, excellent formability and processability. It shows good safety performance, excellent mechanical strength and storability. Therefore, it plays an important role in the military, aerospace and other fields. Studies on the structure–performance of PBX have been paid much attention due to the advantages mentioned above.^1–5^ PBX is a composite material with multi-components so that there is not a specific classification method. Generally speaking, it consists of a body explosive, polymer binders and many other additives.^6^ The most important components in PBX are the body explosive and polymer binders. Common body explosives are cyclotrimethylnitrosamines (RDX),^7,8^ cyclotetramethylenetetramine (HMX)^9,10^ hexanitrohexaazaisowurtzitane (CL-20),^11,12^*etc.* Among these explosives, CL-20 is the most promising candidate due to its better detonation performance than RDX and HMX.^13–18^ Up to now, it has been proved that CL-20 exists as four types at room temperature (298 K): *α*, *β*, *γ* and *ε*. According to previous research, ε-CL-20 showed better stability, lower sensitivity and higher density.^19,20^ The single molecular, single crystal and 4 × 3 × 3 supercell structures of ε-CL-20 are shown in Fig. 1. Due to the higher energy and excellent detonation performance it gradually became a hot issue in the explosive field. Studies on CL-20 have developed for many years, and the further study has shifted from lab^16,21,22^ to practical application.^23^ However, the high sensitivity to initiation of CL-20 cannot be neglected. To improve this drawback, adding polymer into CL-20 and processing PBX is considered to be an efficient method. Therefore, the studies on ε-CL-20-based PBX are significant. Previous studies has proved that (001) crystal surface of CL-20 is the vital growth surface and shows better comprehensive properties.^24,25^ Then in this paper, we chose the CL-20(001) crystal surface as the base explosive system to perform the whole work.

**Fig. 2:**
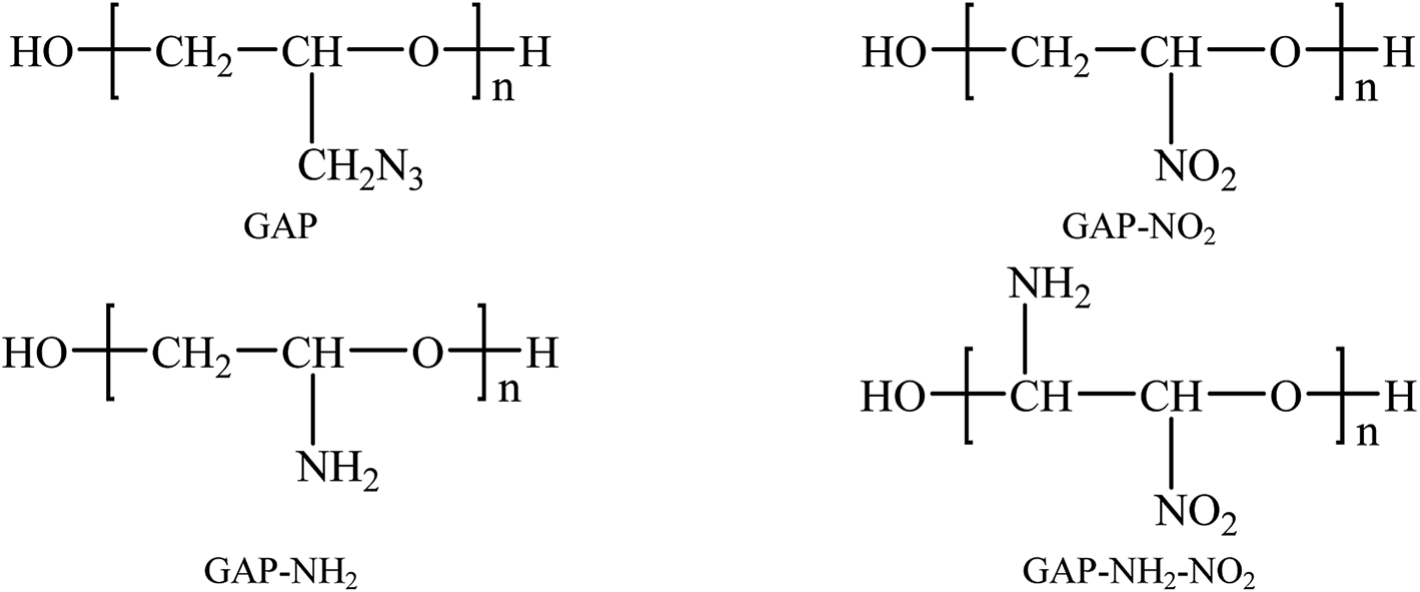
The molecular structures of target polymers.

Common binders used in PBX are thiokol, polyurethane, hydroxyl-terminated polybutadiene (HTPB) and so on.^26–29^ HTPB is more widely used among the binders above. But some shortcomings restrict its further application, such as the inertia and low density. To process PBX with more energetic and higher density, finding a binder with energy and high density is a meaningful task. When comes to energetic binders, azide polymers are representative. Glycidyl azide polymer (GAP) is a typical and common-used energetic binder due to its high density.^30–32^ Then experimental and theoretical researches on the influence of GAP in PBX is a hot issue.^33–35^ As an energetic binder, GAP plays an important role in explosive field. But the poor mechanical properties at low temperature restrict its further application. To improve this shortcoming, we have studied GAP and other three polymers derived from GAP in previous work. They are labeled as GAP-NH_2_, GAP-NO_2_ and GAP-NH_2_-NO_2_. It has been proved that pure GAP-NO_2_ has lowest *T*_g_ and the best comprehensive properties than other polymers.^36^Fig. 2 shows the structures of the four polymers. However, the compatibility and mechanical properties with base explosives as addictive have not been studied yet. As we know, traditional method to study the CL-20-based PBX is experimental research. However, as a kind of high energetic explosive, experimental research is not only dangerous but also costly. To reduce the risk and cost, theoretical research approaches are significant and necessary, such as MD simulation. This method has been widely used to investigate high energetic explosives^37–42^ and the results show good agreement with experimental researches. To perform the whole work more efficiently and safely, molecular dynamic (MD) method was applied.

In this paper, four polymers were chosen to add into the CL-20(001) crystal to build PBX systems and these models were correspondingly abbreviated as CL-20(001)/GAP, CL-20(001)/GAP-NH_2_, CL-20(001)/GAP-NO_2_, CL-20(001)/GAP-NH_2_-NO_2_. They were labeled as A1, A2, A3, A4, respectively, and the pure CL-20(001) system was labeled as A.

## 2. Simulation details

### 2.1 Choice of force field

Choosing a suitable calculation method is significant to the reliability of simulation results. Therefore, the choice of force field plays an important role in the whole work. In this paper, the COMPASS (Condensed-phase Optimized Molecular Potentials for Atomistic Simulation Studies) force field was applied to perform the MD simulations. COMPASS force field represents a technology break-through in force field methods. It is the first *ab initio* force field that enables accurate and simultaneous prediction of gas-phase properties (structural, conformational, vibrational, *etc.*) and condensed-phase properties (equation of state, cohesive energies, *etc.*) for a broad range of molecules^43–45^ and polymers.^46–48^ It is also the first high quality force field to consolidate parameters of organic and inorganic materials.^49–51^ Previous studies on CL-20 and GAP have confirmed that COMPASS force field was applicable to explosive and polymer system.^24,36,52^ Therefore the whole work was carried out under COMPASS force field.

### 2.2 Construction and optimization of models

Firstly, a 4 × 3 × 3 supercell was constructed by Super cell, Symmetry, Build suite, Material Studio software, 2014. The base explosive was ε-CL-20 and the crystal structure document was derived from X-ray diffraction.^53^ The parameters of single cell of ε-CL-20 are: *a* = 8.852 Å, *b* = 12.556 Å, *c* = 13.386 Å. We cleaved the crystal cell along with (0 0 1) surface. Then a vacuum layer of 10 Å was added and a new periodic 3D lattice was constructed. The parameters of 3D lattice. The next step was geometry optimization. The quality of geometry optimization was “Fine”. Algorithm method was “Smart”. Geometry optimization with 10 000 steps was carried out to obtain the final structure. The system with the minimum energy was screened for the next calculation. After pure (0 0 1)/CL-20 system were constructed, other four PBX systems were built by adding different polymer chains into the CL-20(001) lattice. The specific method to build PBX systems are listed below. The first step was building molecular chains of GAP, GAP-NH_2_, GAP-NO_2_ and GAP-NH_2_-NO_2_, respectively. The degree of polymerization was 37. Then optimize every polymer chain and constructing amorphous cell with two chains in it. The length (*a*) and width (*b*) of cell were consistent with CL-20(001) lattice. The next is executing optimization for 5000 steps. To obtain absolutely relaxed polymer models, NVT-MD simulations for 1 ns were executed. Then took out the polymer chains and added into CL-20(001) lattice to build the PBX model. Table S1† showed the original parameters of all systems at 298 K. The original periodic structures of four PBX are as Fig. S1.†

### 2.3 MD simulations

This procedure was conducted by Forcite module, the optimized system was imported as original document for the MD simulations. Firstly, MD simulations of 50 ps were conducted under NVT ensemble, COMPASS force field for further optimization. This procedure aimed at relaxing the PBX systems preliminarily. And then compress the PBXs constantly by changing the length of *c*. Geometry optimization for 20 000 steps and NPT-MD simulations for 300 ps were conducted after every compression until the densities reached to stable values. The structures and parameters of finally equilibrated systems were listed in Fig. S2.†

Comparing Fig. S1 and S2,† it's apparent that the distance between polymer chains and CL-20 molecules decreased. The polymer chains were absorbed on the surface of CL-20(001) crystal. This phenomenon may attribute to the van der Waal's force, hydrogen-bond interaction and electrostatic interaction.

## 3. Results and discussion

### 3.1 Judgment of equilibration

When the system reached to an equilibration, the temperature, total energy, cell length and cell angle can be found to be a relative stable value. These results indicated that the system was in a stable state. Fig. S3a–d† showed the A1's value of temperature, energy, cell length and angle fluctuation at 298 K after NPT-MD for 300 ps. From the four graphs, we can find the temperature fluctuated within 5%, the energy fluctuated within a tiny range. What's more, the cell length and angel are all reaching to relative stable values. These results proofed that the system have reached to an balanced state. The balanced model parameters were listed in Table 1.

**Table 1:** The final parameters of CL-20(001) model (A) and PBX models (A1, A2, A3, A4)

Parameters	A	A1	A2	A3
*a*	35.221	35.957	36.072	35.319
*b*	37.469	38.253	38.374	37.547
*c*	38.441	45.338	42.833	44.421
*α*	90.000	90.000	90.000	90.564
*β*	90.000	90.000	90.000	89.850
*γ*	90.000	90.000	90.000	90.251
Density	2.065	1.876	1.891	1.965
Number of atoms	5184	6078	5856	5856

Comparing the parameters of balanced CL-20 with experimental values, the deviation of all data are within 5%. This indicated that the COMPASS force field and researching methods about this study were applicable. Hence the next researches on PBX systems can be programed by these methods.

### 3.2 Cohesive energy density (CED)

CED is defined as the energy provided by others when removing all molecular interaction of 1 mol. It is a parameter to measure intermolecular force, especially the interaction between groups. Generally speaking, the greater the polarity of groups, the stronger the intermolecular force, and the higher the value of CED. CED is calculated by eqn (1). Xianping Chen^54^ and Manjun He^55^ give the explanation about the relation between polymer and ideal gas. In eqn (1), *H*_v_, *RT* and *V*_m_ stand for the mole vaporization heat, the expansion work when vaporizing, and molar volume, respectively.1
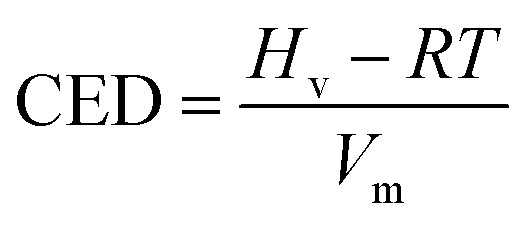


**Fig. 3:**
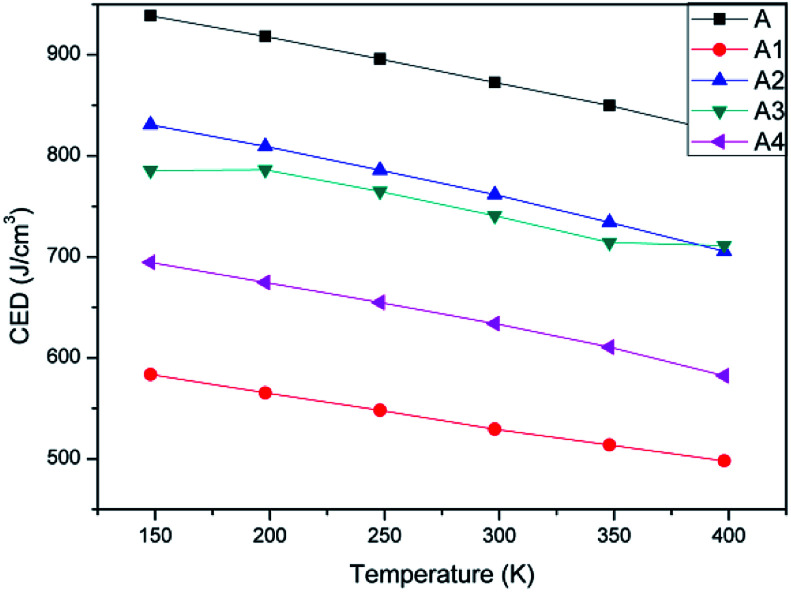
CEDs of A, A1, A2, A3 and A4 as a function of temperature.

The value of CED was mainly decided by the van der Waals force and electrostatic force between molecules in a system. Sometimes it will be affected by hydrogen bond between polar groups. To study the influence of polymer's adding into CL-20 explosive, CEDs of A, A1, A2, A3, and A4 were calculated by cohesive energy density module, Forcite suite. Table 2 showed the CED values and distribution of CL-20(001) and four PBX systems at 298 K. Fig. 3 showed the changing trend of CEDs of five systems as a function of temperature.

**Table 2:** The CEDs and CED distribution of A, A1, A2, A3, A4

Systems	CED (J cm^−3^)	van der Waals (J cm^−3^)	Electrostatic (J cm^−3^)	CED distribution (van der Waals : electrostatic)
A	872.6 ± 0.687	348.1 ± 0.383	512.0 ± 0.576	0.68 : 1
A1	529.4 ± 0.757	129.4 ± 0.435	389.3 ± 0.611	0.33 : 1
A2	761.6 ± 0.770	326.5 ± 0.394	423.6 ± 0.587	0.77 : 1
A3	740.8 ± 0.103	277.3 ± 0.931	451.9 ± 0.102	0.61 : 1
A4	634.0 ± 0.167	181.9 ± 0.105	440.9 ± 0.127	0.41 : 1

Comparing the CED and distribution of system A and other four PBX systems, it can be found that in system A, the rate between van der Waals force and electrostatic force was 0.68 : 1. When polymer chains were added into CL-20(001), the rate was changed apparently. van der Waals force is regarded as relative shorter distance force than electrostatic force. Combining Table 2 and Fig. 3, we can find system A showed the highest CED values than others, this phenomenon may due to the regularity of pure CL-20(001) crystal. The well-defined structure make it difficult to remove the single molecule of CL-20. That's why system A showed high value of CED. Comparing four PBX systems, the addition of polymers destroyed the original structure of CL-20, so that the PBX systems showed lower CEDs than A. Furthermore, we noticed that different groups in polymer affected the CED distribution in different way. In system A1, van der Waals force accounted for a least proportion than other three PBX systems. System A4 showed the slightly higher portion than A1, and A2 showed the highest proportion. In a word, the order of van der Waals' percentage in PBX systems was A1 < A4 < A3 < A2. This order agreed with the order of CED. It indicated that van der Waals force has the major effect on the value of CED. The higher the percentage of van der Waals was, the larger the CED was. The further reason may refer to the strength of hydrogen bond between polymer chains and CL-20 molecules. What's more, according to Fig. 3, we can clearly find that CEDs of all systems decreased with temperature increasing. This result coincided with the fact that the stability becomes poor with temperature increasing.

In sum, adding polymer into CL-20(001) decreased its CED and changed the CED distribution due to the formation of hydrogen bond between polymer and CL-20 molecules.

### 3.3 Mechanical properties

In the energetic materials field, the mechanical properties play a very important role in the manufacture and application. According to elastic mechanics, the stress and strain obey the Hooke's law. The generalized Hooke's law can be described as eqn (2). *C*_*ij*_ in eqn (3) stands for the elastic coefficient. Eqn (3) is the elastic coefficient matrix. The number of elastic coefficients decreased with the symmetry degree increasing of systems. *C*_11_ and *C*_12_ are independent with each other for isotropic material. Therefore, two coefficients *λ* and *μ* called lame coefficient are introduced to simplify the matrix. *C*_12_ is defined as *λ* and *C*_11_–*C*_12_ is defined as 2*μ*. There are four parameters to evaluate the mechanical properties: bulk modulus (*K*), shear modulus (*G*), Young's modulus (*E*) and Poisson's ratio (*γ*). What's more, Cauchy pressure (*C*_12_–*C*_44_) is a parameter to describe the breaking surface feature. The value of *K*/*G* is also regarded as a measurement to evaluate the toughness and ductility in some way. Cauchy pressure and *K*/*G* show similar rule in measuring the deformability. The higher the value, the better the ductility and toughness. Mechanical properties can be the measurement of processability and resistance of explosives to mechanical stress in some way. Good processability and safety are badly needed in terms of PBX. Then explosives that show lower rigidity and higher ductility are desirable. The properties of explosives can be measured by calculations results of mechanical properties. The lower of values of *K*, *G* and *E* are, the better the processability of explosives is. The higher the values of *C*_12_–*C*_44_ and *K*/*G*, the better the safety is. Therefore, the mechanical properties calculation of pure CL-20(001) and PBX systems are necessary. The eqn (4) shows the relationship between *K*, *G*, *E* and *γ*.

2 *σ*_*i*_ = *C*_*ij*_, *ε*_*j*_, *i*, *j* = 1, …, 6

3
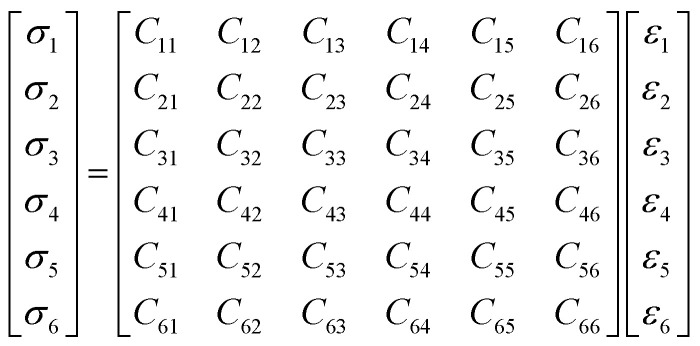
4
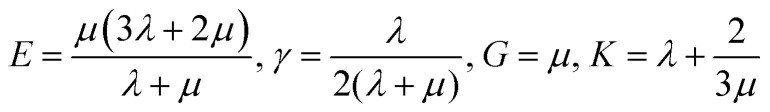


**Fig. 4:**
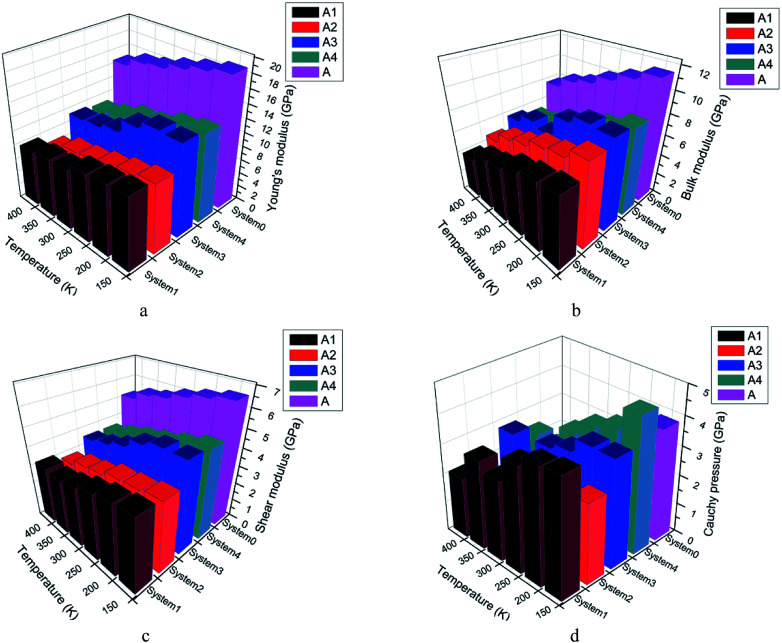
Mechanical properties of system A, A1, A2, A3 and A4.

The mechanical properties of five systems (A, A1, A2, A3, A4) were calculated by mechanical properties suite, Forcite suite. The data in Table S2† were averaged by simulation results of the last 11 frames in PBXs. Mechanical properties of all systems at 248 K were listed in Table S2.† And the changing regularity of mechanical properties as a function of temperature were shown as Fig. 4.

From Table S2† and Fig. 4a–d, we found the pure CL-20(001) system showed the highest Young's, bulk and shear modulus. The number of elastic coefficients were less than 21, which indicated that the pure CL-20(001) and PBX systems were not isotropic. From Fig. 4a–d, we can find the moduli of all systems decreased when the temperature increased, which indicated that the rigidity to resist deformation of these systems decreased and the elasticity were strengthened. Since there is no data from existing literature for the results of PBXs to refer to, we compared the mechanical properties with the pure CL-20(001). Comparing system A and other four systems, it can be found that adding polymers into CL-20 decreased *K*, *G*, *E* of original pure CL-20 system dramatically. For example, the Young's modulus of pure CL-20(001) was 17.24 GPa. It was large and showed a strong rigidity to resist deformation. However, when a small amount of GAP was added into the crystalline surface (001), the Young's modulus decreased to 9.82 GPa, which meant the elasticity of PBX obtained was greatly strengthened. Comparing all systems, the orders of Young's modulus, bulk modulus and shear modulus were A > A3 > A4 > A1 > A2, A > A3 > A2 ≈ A4 > A1 and A > A3 ≈ A4 > A1 ≈ A2, respectively. These results meant that the addition of polymer affected the mechanical properties in a different way. In system A, the structure is regular and well-organized, then it showed high strength and poor toughness. Comparing the four PBX systems, we can find system A3 showed highest elastic, shear and bulk modulus than other three. And system A1 showed the least values. These results can be explained by the hydrogen bonds present in system A3. The interaction between H in CL-20 molecules and O in GAP-NO_2_ offered the strong adhesive force, so that system A3 showed high strength. While in system A1, the huge and stiff pendant group –CH_2_N_3_ in GAP made it difficult to coat the base explosive. That's to say, the compatibility GAP and CL-20 is so poor that the addition of GAP may play a role of defect. Then the PBX showed low strength.

Furthermore, the Cauchy pressure, *K*/*G*, increased apparently according to Table 3 and Fig. 4d when GAP, GAP-NO_2_ and GAP-NH_2_-NO_2_ were added into CL-20, while the addition of GAP-NH_2_ decreased the Cauchy pressure instead. This meant that the addition of polymer changed the breaking surface feature and ductility apparently. Compared with other three PBXs, system A2 owned the least Cauchy pressure. The probable reason may be the hydrogen bond in GAP-NH_2_ itself and the hydrogen bond between polymer chains and CL-20 molecules. The combined factors made system A2 poor strength and ductility.

**Table 3:** N–NO_2_ bond length analysis in different systems (at 298 K)^a^

	A	A1	A2	A3	A4
*L*_ave_	1.3952	1.3949	1.3948	1.3946	1.3951
*L*_prob_	1.3905	1.3955	1.3875	1.3875	1.3905
*L*_max_	1.5485	1.5445	1.5425	1.5245	1.5325

a Remark: the unit of all data is Å.

Then the conclusion that the addition of GAP, GAP-NO_2_ and GAP-NH_2_-NO_2_ into system A improved its rigidity, hardness and the resistance to load impact can be drawn. That's to say, the polymer in PBX can play a role of protection to absorb external shock and acted as a cushion to the base explosive. And system A3 showed better comprehensive mechanical properties. Therefore, the preparation of PBX may be regarded as a method to reduce the sensitivity of the explosive in some way.

### 3.4 Initiation bond length distribution

**Fig. 5:**
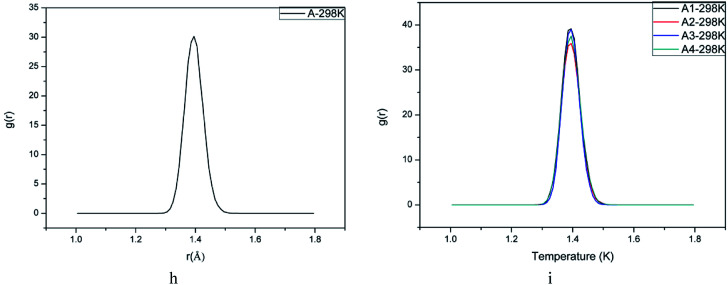
N–NO_2_ bond length distribution in all PBX systems (at 298 K).

**Fig. 6:**
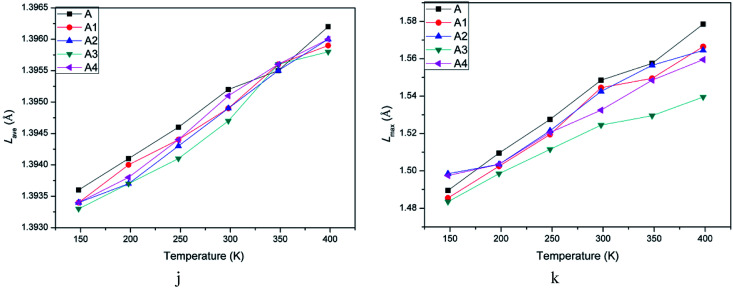
Averaged and maximum bond length of N–NO_2_ as a function of temperature.

The initiation bond is defined as the chemical bond with the lowest energy in an energetic material. The initiation bond is easily triggered with the external influence, and then the explosive occurs. Generally speaking, the shorter the bond length is, the more difficult to break the bond. According to studies about CL-20, we know the initiation bond is N–NO_2_. Therefore, the study on initiation bond changing is significant. In the experimental studies, the averaged bond length can be calculated. But in MD simulations, the initiation bond length distribution and the averaged, maximum and most probable bond length can be calculated by Forcite module. They are labeled as *L*_ave_, *L*_max_, *L*_prob_ for short. Analyzing the change of N–NO_2_ in CL-20 may be helpful to investigate the temperature and polymer's influence on initiation bond. Table 3 showed bond length of CL-20(001) and PBX systems using different statistic method. Fig. 5h and i are the bond length distribution of system A, A1, A2, A3 and A4. Fig. 6j and k are the *L*_max_ of N–NO_2_ as a function of temperature.

According to Fig. 5, it can be found that the bond distribution of all systems showed symmetric Gaussian distribution. The addition of polymers changed the distribution width of N–NO_2_ and increased the probability of *L*_prob_. This indicated that the formation of PBX make the initiation bond more ordered and stable.

From Table 3, it can be obviously found that the addition of GAP, GAP-NH_2_, GAP-NO_2_ and GAP-NH_2_-NO_2_ decreased *L*_max_ and *L*_ave_ slightly. *L*_max_ is the most important parameter among the three. Though the proportion is tiny, the energy is the lowest and easily broken to trigger the decomposition and explosion. Fig. 6j and k showed the changing trend of *L*_ave_ and *L*_max_ when the temperature increased. We can clearly find that *L*_ave_ and *L*_max_ of all systems increased with the temperature rising. It meant that the initiation bond became more and more active with the temperature rising. This phenomenon agreed with the fact that the stability of explosives becomes poor when temperature increases. Then *L*_max_ can be a criterion to measure the sensitivity of explosives. Combing Fig. 6j and k, we can also find that *L*_max_ of pure CL-20 was larger than any other PBX system. And system A3 showed the lowest *L*_max_. Hence we can conclude that the sensitivity of PBX systems was lower than pure CL-20 and adding polymer into explosives to process PBX may be an useful method to obtain more stable explosive. What' more, the addition of GAP-NO_2_ decreased the sensitivity of CL-20 and improved its safety the most obviously. This may attributes to the flexible polymer chain and good ductility of GAP-NO_2_. As a result, it coated the base explosive well and played a role of absorbing impact and heat.

## 4. Conclusion

This paper has programed MD simulations on CL-20(001) and CL-20-based PBXs to study the mechanical properties and sensitivity. The mechanical properties (elastic coefficients, Young's moduli, bulk moduli, shear moduli, Poisson's ratios, Cauchy pressures and *K*/*G*s), CEDs and bond length distributions of pure CL-20(001) and PBXs were calculated. The conclusions are as the following:

(1) CEDs of all systems decreased with the temperature rising, which meant that the stability of them became worse when temperature raised. In addition, adding polymers into CL-20 changed the value of CED and CED distribution obviously. The form of hydrogen bond increased the percentage of van der Waals so that the value of CED increased. Then the order of CED of PBXs was A1 < A4 < A3 < A2.

(2) With temperature rising, the moduli of all systems decreased, which meant the rigidity, brittleness decreased and the elasticity, plasticity were strengthened. In addition, it can also be found that the PBXs owned better ductility and impact resistance than pure CL-20(001) according to the mechanical properties calculation results. System A3 showed highest moduli and relatively better ductility among the four PBX systems. It owned better comprehensive properties.

(3) Studies on initiation bond (N–NO_2_) length distribution indicated the addition of polymer affected *L*_max_ and *L*_ave_ apparently. And the *L*_max_s and *L*_ave_s of system A1, A2, A3 and A4 were shorter than pure CL-20(001), which meant that adding polymer into CL-20 was benefit for reducing its sensitivity. Furthermore, the maximum bond length of N–NO_2_ in system A3 was shorter than other four systems. Then we can regarded that the sensitivity of A3 is lower than other PBX systems.

In all, MD simulation studies on pure CL-20(001) and CL-20(001)-based PBX provided us with useful information of their CEDs, mechanical properties and initiation bond length distribution. This work is meaningful to screen novel polymers for binder used in explosives and measure the comprehensive properties of PBX.

## Conflicts of interest

There are no conflicts to declare.

## Supplementary Material

RA-008-C7RA13517C-s001
