# Supplementary material for: Molecular dynamics simulations on ε-CL-20-based PBXs with added GAP and its derivative polymers
Source: RSC Adv. 2018 Jan 29;8(9):4955–62. doi: 10.1039/c7ra13517c (PMC9077807; doi:10.1039/c7ra13517c)
Supplement: RA-008-C7RA13517C-s001 [file RA-008-C7RA13517C-s001.pdf]

Electronic Supplementary Material (ESI) for RSC Advances.
This journal is © The Royal Society of Chemistry 2018

Supplement Information

ID: RA-ART-12-2017-013517

TITLE: Molecular dynamics simulations on ε-CL-20-based PBXs added by GAP and its derivative polymers

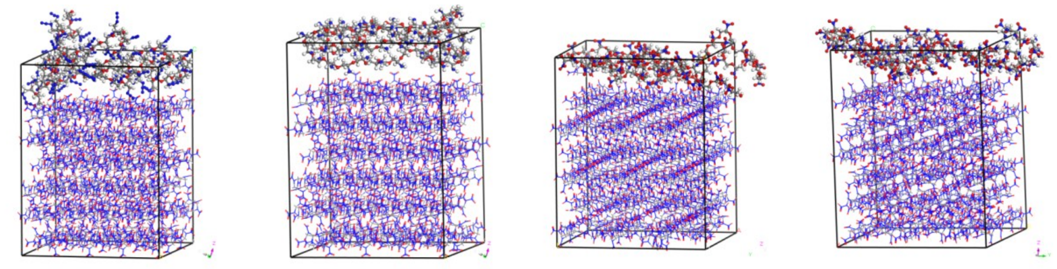

A1 A2 A3 A4

Fig.S1 The original periodic structures of A1, A2, A3, and A4.

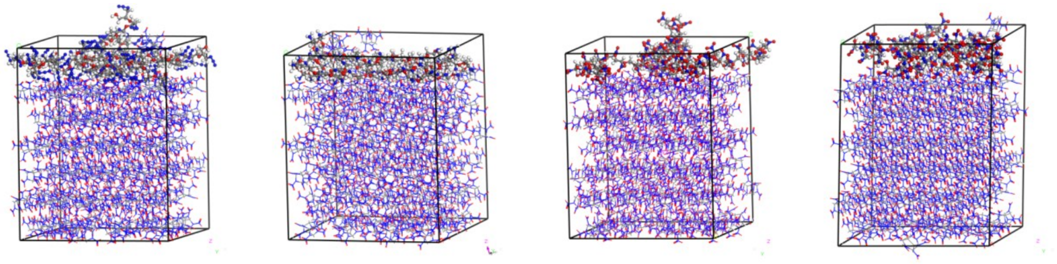

A1’ A2’ A3’ A4’

Fig.S2 The balanced PBX models at 298 K

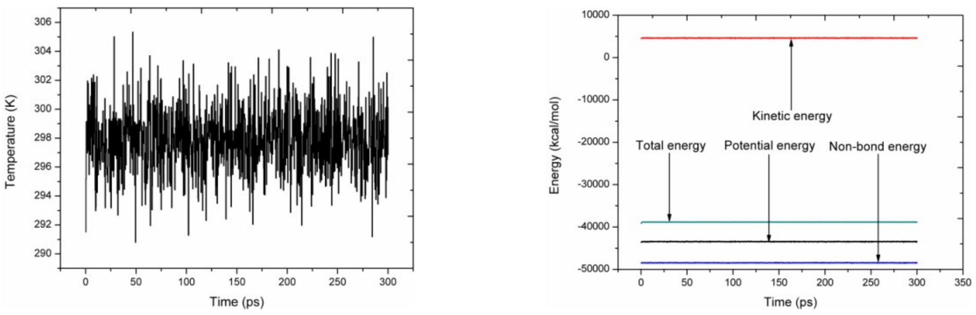

a b

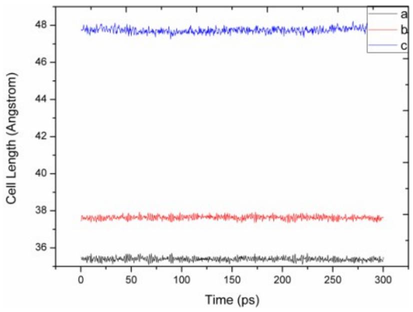

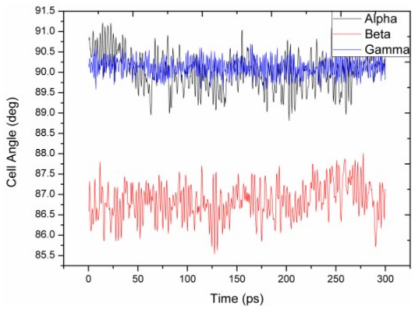

c d

Fig.S3 The balanced parameters of system A1 at 298K

Table S1 Original parameters of CL-20(001) model (A) and PBX models (A1, A2, A3, A4)

Parameters	A	A1	A2	A3	A4
a (Å)	35.408	35.408	35.408	35.408	35.408
b (Å)	37.668	37.668	37.668	37.668	37.668
c (Å)	38.645	48.645	48.645	48.645	48.645
A	90.000	90.000	90.000	90.000	90.000
β	90.000	90.000	90.000	90.000	90.000
γ	90.000	90.000	90.000	90.000	90.000
Density	2.038	1.804	1.728	1.785	1.813
Number of atoms	5184	6078	5856	5856	6004

Table S2 The mechanical properties of PBXs at 248 K

	A	A1	A2	A3	A4
C_{11}	17.99±0.11	13.21±0.12	10.19±0.05	14.93±0.08	14.14±0.09
C_{12}	7.15±0.08	5.91±0.10	4.66±0.03	6.91±0.09	6.72±0.07
C_{13}	5.85±0.10	2.52±0.12	5.31±0.05	4.24±0.07	2.79±0.07
C_{15}	-1.14±0.08	-1.01±0.05	0.19± 0.04	-1.03±0.05	-1.08±0.05
C_{22}	19.08±0.09	13.65±0.08	10.80± 0.04	15.39±0.09	15.67±0.07
C_{23}	0.10±0.06	2.09±0.09	4.02±0.04	2.02±0.06	1.59±0.05
C_{25}	-1.50±0.06	-0.59±0.04	-0.14±0.02	-0.88±0.04	-0.99±0.04
C_{33}	27.93±0.09	9.79±0.13	14.50±0.07	16.68±0.07	11.02±0.07
C_{35}	3.98±0.07	1.00±0.05	1.91±0.05	1.96±0.04	0.82±0.03
C_{44}	4.44±0.03	2.32±0.07	3.19±0.02	3.70±0.02	3.14±0.02
C_{46}	-2.37±0.03	-0.95±0.07	-1.00±0.02	-1.80±0.03	-1.61±0.02
C_{55}	4.93±0.05	2.96±0.03	3.61±0.07	3.87±0.03	3.39±0.02
C_{66}	8.01±0.05	5.86±0.08	5.07±0.04	6.82±0.06	7.38±0.03
E	17.24	9.82	8.64	12.35	11.01
K	10.05	6.19	7.37	8.11	6.77
G	6.09	3.62	6.95	4.68	4.33
γ	0.36	0.38	0.31	0.39	0.39
C_{12}-C_{44}	2.71	3.59	1.46	3.21	3.21
K/G	1.65	1.71	1.92	1.73	1.56

Remarks:

1.The unit of data in table 4 is GPa except Poisson’s ratio and K/G;

2. Other elastic coefficients unlisted are about 0.
